# Correction to “Clinical pharmacokinetics of atropine oral gel formulation in healthy volunteers”

**DOI:** 10.1111/cts.13817

**Published:** 2024-05-06

Parrot M , Yathavan B , Averin O , Hoggard L , Rower JE , Voight M , Greene D , Tarrell A , Whelan A , Ghandehari H , Murphy N , Yellepeddi V . Clinical pharmacokinetics of atropine oral gel formulation in healthy volunteers. Clin Transl Sci. 2024;17(3):e13753. doi:10.1111/cts.13753. PMID: 38465519; PMCID: PMC10926053.38465519 PMC10926053

The author's name “Greene D” is spelled incorrectly. It should be spelled “Green DJ”. Full name is as follows: First—Danielle, Middle initial—J, Last—Green.

We apologize for this error.

